# Pitfalls of AHI system of severity grading in obstructive sleep apnoea

**DOI:** 10.5935/1984-0063.20220001

**Published:** 2022

**Authors:** Rashmi Soori, Nandakishore Baikunje, Ivor D’sa, Navneet Bhushan, Belur Nagabhushana, Giridhar Belur Hosmane

**Affiliations:** 1 K S Hegde Medical Academy, Anaesthesiology and critical care - Mangalore - Karnataka - India.; 2 K S Hegde Medical Academy, Pulmonary Mediicne - Mangalore - Karnataka - India.; 3 K S Hegde Medical Academy, General medicine - Mangalore - Karnataka - India.; 4 Crafitti Consulting private limited, Founder - Director - Bangalore - Karnataka - India.; 5 L-1 ID solutions, IT solutions - Bangalore - Karnataka - India.

**Keywords:** Sleep Apnea, Obstructive, Sleep Apnea Syndromes, Sleep Stages

## Abstract

Obstructive sleep apnoea (OSA) is the major underlying co-morbidity in many of the non-communicable diseases (NCD) due to obesity as a common risk factor. Incidence and prevalence of OSA is on the constant rise ever since this entity came to forefront three decades ago. Precise treatment of underlying OSA is extremely important in major NCDs like diabetes mellitus, hypertension, endocrine disorders and vascular diseases. OSA is subcategorized in to mild, moderate and severe based of apnoea-hypopnea index (AHI). Based on the severity grading, treatment of OSA ranges from life style modifications to oral appliances, continuous positive airway pressure (CPAP) and surgeries. AHI system of severity grading in OSA has several inherent shortcomings and using AHI system for severity grading as the holy grail is likely to be counter-productive. AHI system equates apnoea and hypopnea as equal events, whereas physiological effects vary significantly. AHI system does not account duration of apnoea or body position during apnoeic events. We discuss at length the pitfalls of AHI system of severity grading in OSA.

## INTRODUCTION

Obstructive sleep apnoea (OSA) is a sleep related breathing disorder characterised by narrowing of the upper airway due to either anatomical obstructions such as facial skeletal deformity, hypertrophied adenoid and/or tonsils, macroglossia, micrognathia or due to physiological causes like loss of tone of oropharyngeal musculature during sleep leading to repetitive episodes of apnoea, hypopnoea and/or respiratory effort related arousal (RERA).

As per the American Academy of Sleep Medicine (AASM), OSA diagnosis is made when a person complains of symptoms like snoring, excessive daytime sleepiness, morning headache, awakening with a gasping or choking sensation along with an apnoea hypopnoea index (AHI) of ≥5 or AHI ≥15 without symptoms.^1,2^ Apnoea hypopnoea index is defined as the total number of apnoea and hypopnoea per hour of sleep.

As per the third edition of the International Classification of Sleep Disorders (ICSD-3), Respiratory disturbance index (RDI) is used in the diagnosis of OSA. Respiratory disturbance index (RDI) includes apnoea, hypopnoea and RERA (respiratory effort related arousal) per hour of sleep.^1,2^

## OSA SEVERITY GRADING

As per AASM, OSA severity is graded depending on the AHI. Mild OSA with AHI of 5 to <15, moderate with AHI of 15 to 30 and severe with AHI of >30.^1^ AHI is the only indicator of severity of OSA currently accepted by scientific societies, as it is simple to calculate and it is also objective measure of events such as apnoea and hypopnea.

The AHI is a simple severity grading that counts the number of complete and partial obstructions that occur per hour of sleep. AHI is a reflection of total number of apnoea and hypopnea occurred per hour of sleep. Though AHI system of severity grading is far from accurate it persisted because of the software which simplifies the task of computing the AHI. Since AHI essentially depends on the rate of events captured, it incorporates severity of the individual events only to the extent that event severity correlates with frequency.^3^

## DISCUSSION

Any severity grading from mild to severe usually shows linear correlation with physiological consequences, morbidity or mortality. However, it is not true for OSA severity grading based on AHI score. This is shown in a historical cohort study by Tetyana Kendzerska et al who conducted on 13,438 adults with diagnostic polysomnography and followed up for the occurrence of myocardial infarction, stroke, congestive heart failure, revascularization procedures, or all cause death which measured the composite outcome. They concluded that predictors of composite cardiovascular outcome were OSA-related factors other than AHI.

Independent predictors for complications related to OSA were time spent with oxygen saturation <90%, sleep time, awakenings, periodic leg movements, heart rate, and daytime sleepiness.^4^ A study by Giridhar et al concluded that there was no linear correlation between CPAP titration pressures and severity of OSA.^5^

AHI gives equal weightage to apnoea and hypopnoea. However, apnoea might have higher physiological consequences like severe desaturation and exaggerated autonomic responses compared to hypopnoea. AHI also does not consider the duration of the event while assessing severity. An apnoea lasting for shorter duration (say 10 seconds) may lead to lesser desaturation compared to apnoea of longer duration (say 50 seconds). Longer duration of events may lead to lesser number of events per hour thereby placing the patient in a lesser severity grading as per AHI.

Both these aspects are shown in a study conducted by Antti Kulkas et al. They conducted a study on 395 patients suspected to have OSA who underwent polysomnography. The severity of desaturation related to the events (hypopnea and obstructive apnea) were compared which showed that hypopneas led to less severe desaturation compared to obstructive apneas. Also, longer event duration led to more severe desaturation. While estimating OSA severity and long-term cardiovascular risk, obstructive apneas should have more weightage than hypopneas and event duration should also be considered. However, AHI gives equal weightage to apnoea and hypopnoea. Also, AHI only considers the event without considering the duration of the event.^6^

AHI does not differentiate between hypoxic and non-hypoxic events. Hypoxic events are likely to have profound adverse effects on the autonomic system. This is shown by Koch et al. They evaluated 1022 unique individuals, with 2 112 sleep studies. More than 30% drop in air flow lasting more than 10 seconds was considered as an event. Central events were excluded and was calculated per hour of sleep to say as BDI (breathing disturbance index). BDI was divided into hypoxic or non-hypoxic. Hypertension was found to be more prevalent in subjects with Hypoxic BDI >5. This shows that the effect of each event is more important than the event itself.^7^

Muraja-Murro et al showed that severe health related consequences like mortality in severe OSA were related to longer apnoeas and longer and deeper desaturations. Furthermore, based on multivariate logistic regression analysis, mortality in patients with severe OSA was statistically related to severity of obstructive events. ^8^ In the study by Marin, evidence was given that men with severe sleep apnoea had increased fatal and non-fatal cardiovascular events which could be reduced by CPAP treatment^9^ or oral appliance therapy as suggested by the observational study by Anandam et al.^10^

AHI does not consider the position of sleep at which the events (apnoea/ hypopnoea) occur. Oksenberg et al found that, in severe OSA, the duration of apnoea and the drop in arterial oxyhemoglobin saturation is more in the supine position than in the lateral.^11^ This might lead to lesser event number than the actual severity of the situation while considering AHI.A study by Timo Leppanen et al showed that supine position led to greater number of events compared to the non-supine position. While grading the severity of OSA, the position in which the events occurred should also be paid attention to. ^12^ Bittencout et al showed that night to night variability in AHI is seen in mild and moderate OSA subjects. This could be due to night to night variability in the time spent in supine position.^13^ Kulkas et al opined that in the supine dominant OSA patients, the risk ratios of mortality and cardiovascular morbidity are elevated.^14^

Daytime sleepiness in OSA is inversely attributed to sleep efficiency. AHI does not give any consideration to sleep efficiency. Katherine Cheshire et al, assessed the relationship of sleep disruption, hypoxemia, and mood with cognitive performance and daytime sleepiness in 29 OSA patients. They showed that daytime function in OSA patients is related to nocturnal hypoxemia, sleep disruption and the frequency of breathing irregularities. The patients requiring treatment should be decided based on all these factors.^15^A study by Walter et al found that there was no correlation between AHI severity grading and excessive daytime sleepiness (scored by Epworth sleepiness score).^16^

AHI does not consider the systemic effects of the event. This was shown by Zhang et al. They evaluated 2111 polysomnography data and found that sleep heart rate variability (HRV) particularly the high frequency (HF) component is an independent predictor of cardiovascular disease outcomes. CVD latency correlated with the normalized HF. The proposed prediction model in which sleep HRV served as a supplement to the well-recognized CVD risk factors, such as aging, adiposity and sleep disorders while achieving a total accuracy of 75.3%. This suggests that including heart rate variability in severity grading may predict the actual impact on physiological parameters later leading to pathological changes.^17^ A study by Kulkas et al concluded that novel desaturation severity parameter taking into account the severity and duration of the desaturation events, might be a better predictor of OSA severity than the conventional oxygen desaturation index (ODI).^18^

Any event, apnoea, hypopnoea or RERA (respiratory effort related arousal) lasting less than 10 seconds, however occurring in clusters might have physiological consequences like desaturation, arousal etc; and might have an impact on the health consequence of the individual with OSA^19^.

AHI system of severity grading do not distinguish events based on the sleep stage. There are recent studies which have showed significant differences in physiological effects for apnoea occurring during REM sleep and NREM sleep^20^. These variations are not given weightage in AHI system of severity grading.

Although the argument that AHI reasonably predicts the presence or development of clinical outcomes and hence the measure of disease severity, it is with limitations and should be at best considered an imprecise and crude metric of OSA.^21^

Physiological surrogates such as desaturation, heart rate variability, arousals, sleep stage and body position may serve very well against the reference standard and may do their job well in clinical practice.^21,22^

## CONCLUSION

AHI poorly reflects myriad of physiological complexities occurring as a consequence of reduced airflow, autonomic responses and sleep fragmentation. Development of an algorithm which encompasses physiological effects of airflow limitation for severity grading is desirable and is awaited by further studies.

## References

[1] Kapur VK, Auckley DH, Chowdhuri S, Kuhlmann DC, Mehra R, Ramar K, Harrod CG (2017). Clinical practice guideline for diagnostic testing for adult obstructive sleep apnea: an American Academy of Sleep Medicine clinical practice guideline. Journal of Clinical Sleep Medicine.

[2] Tsara V, Amfilochiou A, Papagrigorakis MJ, Georgopoulos D, Liolios E (2009). Guidelines for diagnosis and treatment of sleep-related breathing disorders in adults and children. Definition and classification of sleep related breathing disorders in adults: different types and indications for sleep studies (Part 1). Hippokratia.

[3] Rapoport DM (2016). POINT: is the apnea-hypopnea index the best way to quantify the severity of sleep-disordered breathing? Yes. Chest.

[4] Kendzerska T, Gershon AS, Hawker G, Leung RS, Tomlinson G (2014). Obstructive sleep apnea and risk of cardiovascular events and all-cause mortality: a decade-long historical cohort study. PLoS medicine.

[5] Giridhar BH, Rajesh V, Soori Rashmi, Rao Anusha, Verma Sanjay Is Severity of Obstructive Sleep Apnoea Is A Surrogate Marker Of Cpap Titration Pressure?.

[6] (2015). NJMR.

[7] Kulkas A, Duce B, Leppänen T, Hukins C, Töyräs J (2017). Severity of desaturation events differs between hypopnea and obstructive apnea events and is modulated by their duration in obstructive sleep apnea. Sleep and Breathing.

[8] Koch H., Schneider L. D., Finn L. A., Leary E. B., Peppard P. E., Hagen E., Mignot E. (2017). Breathing disturbances without hypoxia are associated with objective sleepiness in sleep apnea. Sleep.

[9] Muraja-Murro A., Kulkas A., Hiltunen M. (2013). “The severity of individual obstruction events is related to increased mortality rate in severe obstructive sleep apnea,”. Journal of Sleep Research.

[10] Marin JM, Carrizo SJ, Vicente E, Agusti AG (2005). Long-term cardiovascular outcomes in men with obstructive sleep apnoea-hypopnoea with or without treatment with continuous positive airway pressure: an observational study. The Lancet.

[11] Anandam A, Patil M, Akinnusi M, Jaoude P, El‐Solh AA (2013). Cardiovascular mortality in obstructive sleep apnoea treated with continuous positive airway pressure or oral appliance: an observational study. Respirology.

[12] Oksenberg A., Arons E., Nasser K., Vander T., Radwan H. (2010). “REM-related obstructive sleep apnea: the effect of body position,”. Journal of Clinical Sleep Medicine.

[13] Leppänen T, Töyräs J, Muraja-Murro A, Kupari S, Tiihonen P, Mervaala E, Kulkas A (2016). Length of individual apnea events is increased by supine position and modulated by severity of obstructive sleep apnea. Sleep disorders.

[14] Bittencourt LRA, Suchecki D, Tufik S (2001). The variability of the apnoeahypopnoea index. J Sleep Res.

[15] Kulkas A., Muraja-Murro A., Tiihonen P., Mervaala E., Toyras J. (2015). “Morbidity and mortality risk ratios are elevated in severe supine dominant OSA: a long-term follow-up study,”. Sleep and Breathing.

[16] Cheshire K, Engleman H, Deary I, Shapiro C, Douglas NJ (1992). Factors impairing daytime performance in patients with sleep apnea/hypopnea syndrome. Archives of internal medicine.

[17] Walter TJ, Foldvary N, Mascha E, Dinner D, Golish J (2002). Comparison of Epworth Sleepiness Scale scores by patients with obstructive sleep apnea and their bed partners. Sleep medicine.

[18] Zhang L, Wu H, Zhang X, Wei X, Hou F, Ma Y (2020). Sleep heart rate variability assists the automatic prediction of long-term cardiovascular outcomes. Sleep Medicine.

[19] Kulkas A., Tiihonen P., Julkunen P., Mervaala E., Toyras J. (2013). “Novel parameters indicate significant differences in severity of obstructive sleep apnea with patients having similar apnea hypopnea index,”. Medical and Biological Engineering and Computing.

[20] Shamim-Uzzaman A Q. Afifa, Singh Sukhmani, Chowdhuri Susmita (2018). Hypopnea definitions, determinants and dilemmas: a focused review. Sleep Science and Practice.

[21] Al Oweidat K., AlRyalat S. A., Al-Essa M., Obeidat N. (2018). Comparing REM- and NREM-Related Obstructive Sleep Apnea in Jordan: A Cross-Sectional Study. Canadian Respiratory Journal.

[22] Punjabi NM (2016). COUNTERPOINT: is the apnea-hypopnea index the best way to quantify the severity of sleep-disordered breathing? No. Chest.

[23] Penzel T, Schöbel C, Fietze I (2015). Revise respiratory event criteria or revise severity thresholds for sleep apnea definition. Journal of clinical sleep medicine.

[24] Garg N, Rolle AJ, Lee TA, Prasad B (2014). Home-based diagnosis of obstructive sleep apnea in an urban population. J Clin Sleep Med.

